# Fatal and non-fatal AIDS and non-AIDS events in HIV-1 infected patients with high CD4 counts

**DOI:** 10.1186/1758-2652-13-S4-O39

**Published:** 2010-11-08

**Authors:** J Reekie, J Gatell, I Yust, E Bakowska, A Rachmanova, M Losso, M Krasnov, P Francioli, J Kowalska, A Mocroft

**Affiliations:** 1 University College London, Department of Infection and Population Health, London, UK; 2 Hospital Clinic, Barcelona, Spain; 3 Ichilov Hospital, Tel Aviv, Israel; 4 Centrum Diagnostyki i Terapii AIDS, Warsaw, Poland; 5 Medical Academy Botkin Hospital, St Petersburg, Russian Federation; 6 Hospital JM Ramos Mejia, Buenos Aires, Argentina; 7 Kharkov State Medical University, Kharkov, Ukraine; 8 Centre Hospitalier Universitaire Vaudois, Lausanne, Switzerland; 9 Universtiy of Copenhagen, Copenhagen HIV programme, Copenhagen, Denmark

## Introduction

The risk of uncontrolled viral replication in HIV+ patients who are not immune compromised on the development of serious clinical events is not fully understood. We aimed to compare the incidence of fatal and non-fatal AIDS and non-AIDS events occurring at CD4 counts >350 cells/mm^3^ in different viral load strata (≤500, 501-10000, >10000 copies/ml).

## Methods

Patients contributed person years at risk if the most recent CD4 count was >350 cells/mm^3^ and viral load was measured in the 6 months prior. Poisson regression investigated the relationship between viremia and clinical events, after adjustment for confounding variables.

## Results

**Figure 1:**
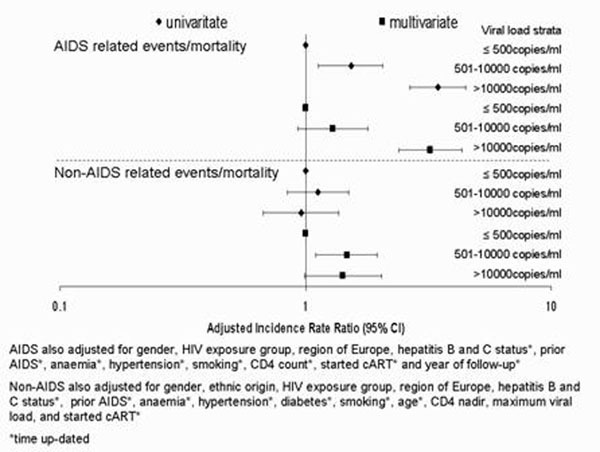


10998 patients were included contributing 43524 person-years of follow-up (PYFU). The majority of follow-up (80%) was with a viral load ≤500, 12% between 501-10000 and 8% >10000. 95%, 72% and 64% of the follow-up in each strata respectively was in patients who had started cART. 379 AIDS events (14 deaths) occurred. There was a lower incidence of AIDS events in patients with a viral load ≤500 (IR 0.69 per 100 PYFU, 95%CI 0.60-0.78) compared to a viral load >10000 (IR 2.38 per 100 PYFU, 95%CI 1.87-2.89). 532 non-AIDS events (131 deaths) occurred. Patients with a viral load ≤500 had an incidence of non-AIDS events of 1.50 per 100 PYFU (95%CI 1.36-1.64), and 1.43 per 100 PYFU (95%CI 0.96-1.89) when viral load >10000. As shown in Figure 1, after adjustment, patients with a viral load >10000 had a 3 times higher incidence of AIDS events than those with a viral load ≤500(p<.0001). In univariate analysis the incidence of non-AIDS events was similar in different viral load strata (p=0.90) but after adjustment, particularly for age and starting cART, there was a 50% and 42% higher incidence of non-AIDS events in patients with a viral load 501-10000 (p=0.008) and >10000 (p=0.05) respectively, compared to a viral load ≤500. The effect of viral load was independent of current CD4 count and was similar in different CD4 count strata (test for interaction p>0.05 for both endpoints).

## Conclusions

In patients with a CD4 count >350 cells/mm^3^ an increased incidence of fatal and non-fatal AIDS and non-AIDS events was found in patients with uncontrolled viral replication, even after adjustment for current CD4 count and use of cART. The association between viral replication and AIDS events was clear and consistent with a biological effect, but with non-AIDS events was less clear without a difference between intermediate and high viral replication.

